# Turning over New Leaves

**Published:** 1891-12-05

**Authors:** 


### Turning Over New Leaves.

### Two English Cardinals.

A perusal of Bishop Creighton's admirable little book on the life and times of Cardinal Wolsey* can hardly fail to suggest to the reader a comparison—and a very interesting comparison we think it is—between its hero and another English Cardinal of the Roman Church who has for many years been prominently before the public, and whose loss will be keenly felt, not merely by that Church and its English adherents, but by Englishmen at large. Never, surely, has a greater difference been seen between two men holding the same office, than that which marks the respective lives of the aged Cardinal who last year celebrated his silver jubilee, and Thomas Wolsey, Abbot of St. Albans, Bishop of Lincoln and Tournai, Archbishop of York, Papal Legate, Cardinal of the Roman Church, candidate for the Papacy, and last, but not least, foremost minister of the Crown.

If it be true—and it must to a great extent be true—that the time makes the man, this will no doubt account in part for the contrast between these two Cardinals. In Wolsey's time, an ecclesiastic and a statesman seem to have been almost interchangeable terms; as Bishop Creighton remarks, "The energy of the Church was placed at the disposal of the State, and was by it absorbed," and the Church's revenues "supplied the King with officials more than they supplied the country with faithful pastors." It was no more than natural, then, that Wolsey should devote himself to politics; for this he had been raised to power, and for this, not for any services, other than political, to the Church, he received the rich and varied rewards with which the list of his titles given above acquaints us.

Now in our own time, as everyone knows, very different things are expected of an ecclesiastic. We will not say that Rome requires no political services of her servants; still, there can be no doubt that the Roman Church of the present day concerns itself (perhaps through force of circumstances only) with temporal things far less than it did in the days of Henry VIII., while it is manifest that even if the English State still expected political services from ecclesiastics, she could not accept them from an ecclesiastic of an alien Church, such as Cardinal Manning is.

But if the times are different, so also are the men. One cannot but feel that, could Wolsey have been transferred to our own time, and given a position such as Cardinal Manning's, he would have shown a very different spirit from that which our present Cardinal shows. Cardinal Manning is, to all appearance, wonderfully free from pride and self-seeking, taking a foremost place when it seems to be pointed out for him, but never thrusting himself into prominence; Wolsey took thought for his own interests as well as for those of his country, and "clung to power even when that power had ceased to be useful for his ends." We do not, indeed, accept Shakespeare's picture of him as a self-seeking politician who, when the Queen pleaded for a remission of taxation, was not ashamed to direct that it should be noised abroad "that through our intercession this revokement and pardon comes"; but that the great Cardinal was ambitious, and loved to figure before the world as a great and powerful personage, cannot be doubted.

Cardinal Manning, again, is an ascetic, as a glance at his spare form and almost fleshless face will show. Report says that he lives almost entirely upon eggs and toast, and stints himself even in this simple diet; and it is plain to everyone that the pomps and vanities of this wicked world are little or nothing to him. Wolsey, on the contrary, lived in great luxury, and loved to travel in royal state, his figure being actually first amid the glittering throng that assembled on the Field of the Cloth of Gold. Cardinal Manning, in fact, is a man to whom spiritual things are incomparably more important, and probably more real, than material things. Wolsey was distinctly a man of the world, to whom the spiritual world was, as Bishop Creighton remarks, "thin and unsubstantial to the last," and to whom, as the record of his last days pathetically shows, all attempts to turn to the spiritual work which he had so long neglected, seemed to him like filling his belly with the husks that the swine did eat.

No wonder, then, that the work of the two great Cardinals should be so very different in its very essence as well as in its results. Each has had the welfare of the English people sincerely at heart—we cannot doubt that; although in Wolsey's case patriotism was mixed with a considerable alloy of self-seeking. But while the dream of one—and a noble dream, too, as far as worldly things go—was to impress upon England a sense of her own importance, to win for her a leading position, and, that position once gained, to make her not only the proud and judicious arbiter of European affairs, but a pattern which all other nations desirous of introducing ecclesiastical and social reforms might copy, the other concerns himself with the spiritual and moral welfare of his countrymen, not, indeed, disdaining to give his earnest attention to social questions, but regarding them (or so we read his action) as means to an end, and striving to secure for Christianity—the spirit of Christ—an entrance into every department of life. It is only natural, then, that with such different ultimate ends in view, the life-work of one Cardinal should present a strong contrast to that of the other.

To emphasise but one point in this contrast. Wolsey worked for the English people *en masse*, caring little for the distress of certain classes, if only he could get together money enough to advance the schemes of his royal master and himself, and thus incurring the undying hatred of the populace. Cardinal Manning, on the contrary, espouses the cause of the poor and helpless, thereby winning for himself a love and respect which it is truly wonderful to behold in a country but lately so rampant in its Protestantism.

Then, as to the success of the two Cardinals. As we think of Wolsey's end we can but exclaim, "Oh, what a fall was there, my countrymen!" Hated by the people, deserted by his King, snatched from his proud position before he had even inaugurated the reforms towards which he had been for ever aiming, he died, at a comparatively early age, a broken-down and broken-hearted old man. Cardinal Manning, on the other hand, will leave this world full of years and—who can doubt it?—full of honours. True, it can hardly be said of the populace as of Nature, that it "never did betray the heart that loved it"; but it is rare indeed for a man who has devoted himself with a single heart to the service of others, and has used none but good and lawful means in that service, to find himself deserted at the last. Does not the life of Wolsey afford fresh confirmation of a truth which must have been pressed home to many of us by recent events—that "doing evil that good may come" is not a safe, any more than it is a right, policy; and that he who descends to crooked ways and questionable associates often finds that the staff on which he has been leaning has gone into his hand and pierced it?

### Religious Tract Society's Publications.

It is unfortunately true that the friends most solicitous of our good, and who minister to our wants in an unostentatious manner, often become a mere matter of course to us, and we award them not the thanks which is their due. The Religious Tract Society is an old friend of ours, and we are therefore almost ashamed to admit that we felt any surprise when the complete nature of their periodicals was brought to our notice. Nevertheless, surprise and admiration we certainly felt, on looking at the collection of publications before us. "The Leisure Hour," "The Sunday at Home," "The Boys' Own Paper," and "The Girls' Own Paper," are familiar household friends. But besides these, we find literature of healthy tone and interest for every grade and age in life. The children may well be content, for they have offered them, besides the old favourites already mentioned, admirable supplements in the shape of "In-door Games and Recreations," "Out-door Games and Recreations," "The Girls' Own Out-door Book," and "The Girls' Own In-door Book," all full of interest to boys and girls, and issued in monthly parts. The little ones in the nursery are provided with "The Child's Companion" and "Our Little Dolls," for the tiny ones. "Friendly Greetings," "The Tract Magazine," and "The Cottager and Artisan," contain interesting and healthy literature, and will, we hope, prove successful rivals of the penny dreadfuls and such unprofitable stuff. The Religious Tract Society is a faithful friend to the public, always on the watch to provide its needs in a beneficial manner, and we wish it all the recognition it deserves.

---

An accident with disastrous consequences is reported to have taken place some time ago in a chemist's shop in Berlin. The chemist, in making up a prescription containing chromic acid and alcohol, added the crystalised chromic acid directly to the alcohol. The result was a violent explosion, which destroyed his eyesight.

